# Seminal Plasma Extracellular Vesicles: Key Mediators of Intercellular Communication in Mammalian Reproductive Systems

**DOI:** 10.3390/vetsci12060585

**Published:** 2025-06-13

**Authors:** Yanshe Xie, Chen Peng, Jiayi He, Zhengguang Wang, Jizhong Xiang

**Affiliations:** 1 Hainan Institute, Zhejiang University, Yongyou Industry Park, Yazhou Bay Sci-Tech City, Sanya 572000, China; 12217021@zju.edu.cn (Y.X.); 22217072@zju.edu.cn (C.P.); 12417013@zju.edu.cn (J.H.); 2 College of Animal Sciences, Zhejiang University, Hangzhou 310058, China; 3 Huzhou Miemie Sheep Husbandry Co., Ltd., Huzhou 313023, China

**Keywords:** seminal plasma, extracellular vesicles, infertility, sperm maturation, sperm capacitation, embryo implantation

## Abstract

Seminal plasma, traditionally regarded as a passive transport medium for sperm, has emerged as a sophisticated biofluid orchestrating critical dialogues in reproductive physiology. Contemporary research reveals its multifunctional role in modulating endometrial receptivity through molecular priming of the female reproductive tract, a process essential for successful embryo implantation. Notably, seminal plasma contains numerous extracellular vesicles (EVs) that serve as critical mediators of intercellular communication via the regulation of biological processes in target cells. Through this sophisticated vesicular communication system, seminal plasma extracellular vesicles (SPEVs) coordinate critical reproductive events. Thus, it will be important to elucidate the molecular mechanisms by which SPEVs mediate reproductive processes, to provide knowledge that may improve fertility outcomes. Herein, we elucidated the emerging potential of SPEVs as non-invasive biomarkers for male fertility assessment and infertility diagnosis. Furthermore, this review systematically summarized current advances in SPEVs, highlighting their multifaceted roles in mediating sperm maturation, regulating sperm capacitation, and modulating embryo implantation through targeted delivery of bioactive signaling molecules.

## Simple Summary

Seminal plasma extracellular vesicles have emerged as pivotal mediators of intercellular signaling within the mammalian reproductive system by delivering bioactive signaling molecules to target cells. This review summarizes the emerging roles of seminal plasma extracellular vesicles as non-invasive biomarkers for male fertility assessment and infertility diagnosis, while elucidating their regulatory effects on sperm maturation, sperm function, and embryo implantation. The findings highlight the paramount importance of elucidating the molecular mechanisms through which seminal plasma extracellular vesicles mediate intercellular communication within reproductive systems. Such investigation is essential for improving breeding rate and paving the way for novel therapeutic strategies targeting idiopathic infertility.

## 1. Introduction

The elevated incidence of early gestational failure represents a significant challenge in both livestock and clinical reproduction, with impaired embryo implantation accounting for the majority of pregnancy loss [1,2]. Since its clinical inception four decades ago, in vitro fertilization has catalyzed revolutionary advancements in assisted reproductive technology, fundamentally improving breeding rate and transforming fertility treatment through innovative techniques including artificial insemination (AI), gamete manipulation, intracytoplasmic sperm injection, and precision embryo transfer protocols [3]. Despite these achievements, the paternal contribution to reproductive abnormalities remains largely overlooked [4,5], primarily due to the persistent misconception that sperm merely function as genomic vectors for paternal DNA delivery.

Seminal plasma, a complex bioactive fluid synthesized through coordinated contributions from the testis, epididymis, vas deferens, and accessory glands (notably seminal vesicles and prostate), forms the microenvironment surrounding ejaculated sperm [6]. Emerging research has revealed that sperm delivery to the oocyte during conception represents only one facet of its biological functions. Current scientific evidence establishes that seminal plasma contains bioactive signaling molecules that exert significant regulatory effects on key reproductive processes, including sperm maturation, sperm capacitation, and embryo implantation. During sperm transit through the female reproductive tract (FRT), seminal plasma plays a critical regulatory role in coordinating key reproductive processes: (1) maintaining sperm in a decapacitated state until optimal fertilization timing; (2) facilitating intravaginal sperm transport; (3) establishing the oviductal sperm reservoir; (4) triggering precisely timed acrosome reactions; and (5) modulating gamete interactions through zona pellucida-binding proteins [7]. In addition, following seminal plasma exposure, cervical and uterine epithelial cells upregulate the secretion of proinflammatory cytokines (including GM-CSF, IL-1β, IL-6, and IL-8), which mediate the recruitment of neutrophils, macrophages, and dendritic cells (DCs) from peripheral circulation to both the endometrial stromal compartment and epithelial layer. This coordinated immune response facilitates endometrial receptivity establishment through stromal remodeling while simultaneously creating an immune-privileged microenvironment that safeguards sperm from immunological clearance [8]. However, the molecular mechanisms underlying the regulatory functions of bioactive signaling molecules in seminal plasma within the reproductive system require further elucidation.

Extracellular vesicles (EVs) are nano-sized, lipid bilayer-enclosed particles released by cells that transport bioactive signaling molecules such as proteins, lipids, and nucleic acids [9]. Notably, seminal plasma was among the earliest biological fluids where EVs were detected and characterized [10]. As critical mediators of intercellular communication, EVs facilitate the transfer of these molecules through both direct receptor stimulation and intracellular cargo delivery, thereby modulating cellular functions and signaling pathways [11]. This unique capacity makes EVs pivotal targets for elucidating molecular mechanisms in biological processes. Therefore, this review synthesizes current evidence regarding seminal plasma extracellular vesicles (SPEVs) as non-invasive biomarkers for male infertility diagnosis, with particular emphasis on their cargo characterization and functional roles within reproductive systems, thereby highlighting critical knowledge gaps to guide future investigations.

## 2. Overview of Seminal Plasma Extracellular Vesicles

Initial observations of EVs biogenesis emerged from studies on reticulocyte maturation in 1987 [12]. Since then, EVs were detected across diverse biological fluids, with seminal plasma standing out as one of the earliest biofluids where EVs were systematically characterized in humans [10]. Notably, SPEVs have also been comprehensively investigated across multiple domestic animal species, including bull [13], boar [14], buffalo [15], ram [16], equine [17], and rabbit [18]. The biogenesis of SPEVs initiates with the endocytic uptake of extracellular molecules, leading to the formation of multivesicular bodies (MVBs). These MVBs selectively incorporate proteins, RNAs, and lipids derived from both the cytosol and trans-Golgi network through inward budding of the endosomal membrane, thereby generating intraluminal vesicles (ILVs). Subsequently, these ILVs are released into the extracellular space as SPEVs through exocytosis, a process mediated by the fusion of MVBs with the plasma membrane.

It is worth mentioning that seminal plasma contains a significantly higher concentration of EVs compared to most other bodily fluids, underscoring their critical functional roles in reproduction. In addition, these EVs exhibit heterogeneous origins, primarily derived from the coordinated secretion of various organs in the male reproductive system: specifically, prostate (known as prostasomes) and epididymis (epididymosomes), along with seminal vesicles, ductus deferens, and testicles [19]. SPEVs secreted by testicles and epididymis actively engage with sperm throughout their maturation journey. These vesicles mediate critical molecular transfers of lipids, proteins, and RNAs that are indispensable for developing essential functional competencies, including progressive motility acquisition and fertilization potential [20,21,22]. At ejaculation, the population of SPEVs becomes more heterogeneous, with new EVs being shed from the ductus deferens and specific accessory sex glands. While EVs originating from specific segments of the male genital tract, such as epididymosomes and prostasomes, have been relatively well-characterized, significant gaps persist in our understanding of SPEV heterogeneity and functional specialization across the diverse reproductive tissues. Furthermore, studying SPEVs derived from tissues like the seminal vesicles and ductus deferens has been hampered by their low abundance and the current lack of reliable tissue-specific biomarkers for accurate identification based on origin. Notably, several single-EV profiling technologies have emerged in recent years, including droplet barcode sequencing for protein analysis (DBS-Pro), a novel method enabling high-resolution characterization of surface proteins on individual EVs. This approach facilitates the identification of EV subtypes both within and across samples. In the present study, DBS-Pro was employed to profile multiple surface proteins of single EVs derived from the non-small cell lung cancer (NSCLC) cell line H1975 and clinical samples of NSCLC patients [23]. With a remarkably low mixing rate (<2%), the results demonstrate the method’s capability for robust single-vesicle surface protein profiling and subsequent EV subtype classification. This advancement holds significant potential for advancing the field of SPEV research by elucidating the cellular origins of vesicles.

Overall, the concerted mixture of SPEVs exerts specific effects on sperm capacitation and fertilizing capacity, as well as on the female’s immune response to paternal antigens and the establishment of endometrial receptivity. Moreover, the protection against degradation and functional loss provided by EV encapsulation preserves bioactive molecules more effectively than their free state within seminal plasma. This preservation mechanism is critical for enabling the application of specific EV components as non-invasive biomarkers.

## 3. Harnessing Seminal Plasma Extracellular Vesicle Contents as Non-Invasive Biomarkers for Livestock Fertility Assessment and Male Infertility Diagnosis

In livestock production, AI technology has been extensively utilized for livestock breeding improvement [24]. The precise evaluation and selection of high fertility male livestock for insemination are critically important to enhance conception. However, contemporary evaluation of male livestock fertility predominantly relies on semen quality assessment. Current research indicates that the diagnostic accuracy of these conventional techniques remains suboptimal [25], which significantly limited the application of AI. Within reproductive medicine, azoospermia and oligoasthenoteratozoospermia are recognized as predominant causes of male infertility, the underlying male etiology remains undetermined in approximately 70% of infertile couples [26]. In addition, prostate cancer, the second most prevalent malignancy in men worldwide, poses a significant threat to men’s health and quality of life. According to 2020 global estimates, this disease claimed 375,304 lives worldwide [27]. This deficiency stems partly from the lack of reliable non-invasive diagnostic tools. Current clinical practice predominantly relies on tissue biopsy analyses, which faces significant limitations due to tissue heterogeneity and inherent challenges in sampling techniques, frequently yielding inconclusive results. In this context, the identification of specific non-invasive biomarkers represents a critical priority in both accurate selection of livestock with high fertility and advancing therapeutic strategies for male infertility.

Since EVs have a lipid bilayer structure, the proteins and nucleic acids they carry are stable in body fluids [28]. Additionally, EVs contain molecules of the progenitor cell, so these EVs in the fluids can reflect the identity, characteristics, and health of the cell or tissue of origin [19]. Due to these attributes, the contents of EVs are considered relevant for study as reliable biomarkers. Specifically, in recent years, an increasing number of studies have been published that evaluate the contents of SPEVs in fluids as non-invasive biomarkers for livestock fertility assessment and male infertility diagnosis (Table 1).

Notably, accumulating evidence has demonstrated that non-coding RNAs (ncRNAs) are emerging diagnostic biomarkers due to their crucial biological significance through transcriptional and post-transcriptional modifications [29]. Among them, circRNA exhibits multifaceted regulatory functions in cellular processes, including regulating transcription, promoting DNA breaks, inhibiting RNA binding protein activity, acting as an enhancer or scaffold for various proteins, being translated into functional peptide, and interfering with mRNA function [30]. In addition, circRNA serves as competitive endogenous RNA (ceRNA) to sponge miRNA, forming regulatory networks with lncRNA and mRNA [31,32]. Since this ceRNA network involves multiple RNAs, it provides a multidimensional framework for elucidating complex biological processes in reproductive biology. Thereby, in-depth investigation of ceRNA network dynamics in SPEVs holds promise for improving fertility assessment in livestock production and advancing diagnostic strategies in male infertility. In addition, further research on the functional impact of these biomarkers on mammalian reproductive systems would advance both livestock breeding and clinical applications. However, current studies remain limited, with most efforts focused on identifying novel biomarkers using high-throughput sequencing. The practical feasibility of these biomarkers still requires validation in large populations. Future work should prioritize elucidating their mechanistic roles in reproduction and confirming their diagnostic accuracy at scale, ultimately improving fertility outcomes in animals and humans.

**Table 1:** Summary of seminal plasma extracellular vesicle contents as biomarkers for livestock fertility assessment and male infertility diagnosis in last five years.

Phenotype	Species	Subtype	Biomarker	Reference
Fertility	bull	protein	SP10, ADAM7, and SPAM 1	[33]
		miRNA	miR-195	[34]
	boar	protein	EZRIN *	[35]
		miRNA	miR-26a *	[36]
	buffalo	protein	PDIA4 and GSN	[37]
	rabbit	miRNA	miR-190b-5p, miR-193b-5p, let-7b-3p, and miR-378-3p	[18]
Sperm motility	boar	gene-lipid linkages	CerG1 (d22:0/24:0)-RCAN3, Cer (d18:1/24:0)-SCFD2, and CerG1 (d18:0/24:1)-SCFD2	[38]
		protein	GART, ADCY7, and CDC42	[39]
		miRNA	miR-122-5p, miR-486, miR-451, miR-345-3p, miR-362, and miR-500-5p	[40]
		miRNA	miR-205, miR-493-5p, and miR-378b-3p	[41]
		miRNA	miR-222 *	[42]
		circRNA	circCREBBP *	[43]
	buffalo	protein	ACRBP, SPACA1, PRDX5, SPACA4, DYNLL2, ZAN, IZUMO1, and ADAM2	[15]
Conception rates	boar	protein	GPX5 *	[44]
Semen quality	human	protein	LTF, CRISP3, SERPINA3, ELSPBP1, GSTM3, AGP2, SAP, ANPEP, MME, and FAS	[45]
		miRNA	miR-10b-3p, miR-122-5p, miR-205-5p, miR-222-3p, miR-34c-5p, miR-509-3-5p, miR-888-5p, miR-892a, miR-363-3p, miR-941, miR-146a-5p, and miR-744-5p	
		miRNA	miR-7110, miR-4800, miR-4488, miR-3916, and miR-4508	[46]
		circRNA	hsa_circ_0009013, hsa_circ_0123184, hsa_circ_0114168, hsa_circ_0139507, and hsa_circ_0139505	
		piRNA	piR-hsa-26399, piR-hsa-28160, piR-hsa-28478, and piR-hsa-1077	
		rRNA	URS00008C6BF7, URS00008C9E2E, URS0000914753, URS0000CA0D60, and URS00008CE4BC	
		lncRNA	URS0000D56E09, URS0000D5AE24, URS0000A7764F, ENST00000631211.1, and ENST00000629969.1	
Live birth rate	human	circRNA	hsa_circ_0103367, hsa_circ_0008611, hsa_circ_0008109, hsa_circ_0004177, hsa_circ_0009684, hsa_circ_0013829, hsa_circ_0035429, hsa_circ_0114168, hsa_circ_0001488, and hsa_circ_0118471	[47]
		piRNA	piR-hsa-28478 and piR-hsa-1077	
Azoospermia	human	miRNA	miR-10a-5p, miR-146a-5p, miR-31-5p, miR-181b-5p	[48,49]
Non-obstructive azoospermia	human	tsRNA	tRF-Val-AAC-010 and tRF-Pro-AGG-003	[50]
Oligoasthenospermia	human	circRNA	has_circ_0004721, has_circ_0002452, has_circ_0079245, has_circ_0005584, has_circ_0003823, has_circ_8826, has_circ_0125759, has_circ_0109282, and has_circ_0009142	[51]
Spermatogenic ability	human	piRNA	piR-has-61927	[52]
		protein	ANXA2 and KIF5B	[53]
Unilateral varicocele	human	miRNA	miR-210-3p	[54]
Prostate cancer	human	protein	KLK3, KLK2, MSMB, NEFH, PSCA, PABPC1, TGM4, ALOX15B, and ANO7	[55]
		protein	CRP and H2B2E	[56]
		mRNA	*CASP3*, *DDX11*, *DLC1*, *ETV1*, *PTGS1*, *TP53*, and *VEGF*	
		miRNA	miR-141-3p	
		miRNA	miR-27a-3p, miR-27b-3p, miR-155-5p, and miR-378a-3p	[57]
		tsRNA	5′-tRNA-Glu-TTC-9-1_L30 and 5′-tRNA-Val-CAC-3-1_L30	[58]

* indicates that its functional regulatory mechanisms within the mammalian reproductive system have been experimentally validated in the existing literature.

## 4. Seminal Plasma Extracellular Vesicles Promote Sperm Maturation

Mammalian sperm exhibit transcriptional and translational quiescence due to their highly condensed chromatin structure [59], indicating that post-testicular maturation events in the epididymis and female reproductive system through external signals such as SPEVs are particularly important.

During epididymal maturation, sperm migrate through the three functionally distinct epididymal segments (caput, corpus, and cauda), each exhibiting unique transcriptional and proteomic profiles that drive region-specific sperm remodeling [60,61]. The caput epididymis maintains the most abundant and diverse secretory profile, where a dynamic molecular exchange occurs: testicular-derived proteins from sperm undergo rapid absorption while epididymal-specific proteins are actively secreted. This sophisticated molecular reprogramming ultimately endows sperm with two critical functional competencies: swimming in a progressive manner and the capacity for oocyte recognition [62]. These functional characteristics progressively mature in the corpus epididymis before attaining their peak functional capacity for motility and fertilization in the distal caudal segment [63]. In this process, sperm undergo extensive physiological remodeling mediated through the transfer of proteins and lipids via epididymosomes, including progressive sphingomyelin accumulation and cholesterol depletion [64], membrane rigidity reduction [65], spatial redistribution of surface antigens [66], structural stabilization through increased disulfide bond formation [67], and coordinated surface protein modification through selective removal, addition, and post-translational processing [68].

Epididymosomes constitute a relatively small proportion of SPEVs in ejaculated semen, indicating their primary functional role in sperm maturation during epididymal transit rather than post-ejaculation [69]. These vesicles exhibit a polydisperse size distribution (25–300 nm) with membrane enriched in cholesterol–sphingomyelin lipid rafts that are essential for protein transfer between epididymosomes and sperm. Epididymosomes are proposed to exist as two distinct subpopulations: epididymal sperm binding protein 1 (ELSPBP1)-enriched epididymosomes and CD9-positive epididymosomes. ELSPBP1-enriched epididymosomes are believed to protect epididymal sperm from oxidative stress through an antioxidant cycle. Specifically, these specialized vesicles form a functional complex with biliverdin reductase A (BLVRA), catalyzing the NADPH-dependent reduction of biliverdin to bilirubin. The biliverdin acts as an endogenous antioxidant by effectively scavenging reactive oxygen species (ROS) from immature sperm, thereby protecting maturing sperm. Simultaneously, bilirubin undergoes Zn^2+^-dependent reconversion to biliverdin, completing a redox cycle that sustains antioxidant defense mechanisms [70]. CD9-positive epididymosomes are postulated to orchestrate critical mammalian sperm maturation processes during epididymal transit. It exhibits temperature- and pH-dependent binding and fusion with sperm, mediating targeted protein delivery to post-acrosomal sheath and midpiece domains. This process facilitates mammalian sperm maturation through multiple mechanisms: (1) regulation of Ca^2+^ channel gating, (2) enhancement of zona pellucida binding affinity, (3) activation of progressive motility, and (4) suppression of premature acrosome reaction [71].

Mechanistically, epididymosomes mediate intercellular communication by delivering a heterogeneous population of small non-coding RNAs (sncRNAs), including miRNAs and tRNAs, to maturing sperm. This transfer dynamically remodels the sperm sncRNA profile, potentially regulating post-transcriptional gene expression and contributing to paternal epigenetic inheritance during post-testicular maturation [72,73]. In addition, these sncRNAs are subsequently translocated into the oocyte during gamete fusion, where they establish epigenetic regulation of embryo development through modulation of a specific subset of genes [72]. These findings elucidate the multifaceted roles of epididymosomes in mammalian reproduction, delineating the molecular mechanisms underlying cargo delivery to recipient cells (oocytes or endometrial epithelial cells) and providing mechanistic insights into their regulatory functions during embryo development and implantation processes. However, research progress has been limited by technical challenges in isolating high-purity epididymal sperm and epididymosomes. Notably, epididymosomes exhibit regional heterogeneity in both size and molecular composition along the epididymal segments [74], further complicating their isolation. Recent methodological advancements, including the development of novel Tissue EV isolation protocols and the establishment of animal models for comparative biomarker discovery, show potential to significantly advance this field. Specifically, Yu et al. developed an immunomagnetic separation strategy to selectively purify Tissue EVs by depleting undesired non-Tissue EVs with antibody-conjugated magnetic microparticles [75]. Furthermore, Luo et al. established a method for isolating tissue-specific EV subpopulations by using flow cytometry sorting technology based on membrane surface protein markers [76]. These emerging methodologies could facilitate systematic characterization of molecular mechanisms by which epididymosomes mediate post-testicular sperm maturation processes.

## 5. The Regulatory Role of Seminal Plasma Extracellular Vesicles in Sperm Function

In livestock reproduction, particularly in semen cryopreservation and AI, seminal plasma is traditionally removed or hyper-diluted post-ejaculation. However, research over the past 20 years shows that preserving or reintroducing seminal plasma is crucial for maintaining post-thaw fertility comparable to natural mating, beyond just optimizing sperm quality. Notably, studies have shown that boar post-thaw sperm viability is best restored when seminal plasma is reintroduced [77]. Of note, supplementing cryopreserved sperm from “poor freezer” individuals with seminal plasma derived from “good freezer” males has been shown to enhance cryo-survival rates in both boar [78] and stallion [79]. Furthermore, homologous seminal plasma supplementation during AI has been associated with improved reproductive outcomes, including early embryo survival rate [80], implantation rate [81], placental development [82], farrowing rates, litter size, and even offspring development and health [83]. These collective findings provide compelling evidence for the functional significance of seminal plasma and its EVs in post-ejaculatory reproductive processes.

Following ejaculation, SPEVs exert multifaceted regulatory effects on sperm viability and function through both direct and indirect mechanisms. Direct regulation manifests through their involvement in critical physiological processes including sperm motility enhancement, capacitation initiation, and acrosome reaction, while indirect protective functions are achieved through microenvironmental stabilization for sperm within the FRT.

Sperm motility constitutes a fundamental determinant of natural fertility, particularly for ensuring successful post-ejaculatory survival and functionality within the FRT. SPEVs play a pivotal role in regulating sperm motility, primarily mediated through the transfer of Ca^2+^-signaling receptors to the neck region of ejaculated sperm [84]. Mechanistically, SPEV fusion delivers three critical components to sperm: (1) progesterone receptors, which directly activate the cation channel of sperm (CatSper) to induce Ca^2+^ influx; (2) cyclic adenosine diphosphoribose (cADPR)-synthesizing enzymes, essential for generating Ca^2+^ mobilization second messenger; and (3) ryanodine receptors (RyRs), a family of intracellular Ca^2+^ release channel proteins [84,85]. Beyond Ca^2+^ signaling, emerging evidence indicates that SPEVs enhance sperm progressive motility parameters by providing bioenergetic support through ATP synthesis [86]. In addition, advancements in bioinformatic analysis and next-generation sequencing platforms have catalyzed groundbreaking discoveries in SPEV research, particularly regarding their molecular cargo and functional implications for sperm motility. Specifically, in porcine models, GPX5 significantly enhances sperm motility through elevation of the total antioxidant capacity of sperm [44], while miR-222 exhibits pronounced motility-promoting effects via targeted suppression of BCL2L11-mediated apoptotic pathways [42]. Furthermore, circCREBBP improves sperm motility via the PI3K–Akt signaling pathway through competitive binding miR-10384 and miR-143-3p [43].

Sperm capacitation serves as a critical physiological prerequisite for successful fertilization, during which sperm acquire the ability to undergo the acrosome reaction—an exocytotic event essential for zona pellucida penetration and subsequent fusion with the oocyte plasma membrane [87]. Notably, the timing of this process must be tightly regulated within the FRT, as premature or dysregulated capacitation may lead to premature acrosome reaction and subsequent sperm degeneration, ultimately compromising fertilization potential [88]. SPEVs participate in this process also via the transfer of Ca^2+^-signaling receptors, which orchestrate intracellular Ca^2+^ elevation in sperm. This precisely regulated Ca^2+^ signaling modulates flagellar hyperactivation, thereby underpinning the sperm’s fertilization competence essential for successful gamete fusion [89]. Additionally, SPEVs facilitate sperm capacitation via cAMP-dependent activation of protein kinase A (PKA), which promotes tyrosine phosphorylation [90]. Emerging evidence suggests that SPEVs inhibit sperm capacitation through selective packaging of bioactive cargo, including cytoskeletal protein EZRIN [35] and ncRNAs such as miR-21-5p in porcine models [91]. Although the direct regulatory effects of SPEVs on sperm capacitation have been extensively documented, conflicting findings persist in this research domain. Clinically relevant studies by Pons-Rejraji et al. [92] identified a transient upregulation of tyrosine phosphorylation in sperm proteins during SPEVs treatment, paradoxically culminating in partial capacitation inhibition following prolonged incubation (3 h). This biphasic regulation contrasts with clinical reports by Bechoua et al. [93], who documented sustained downregulatory effects on tyrosine phosphorylation patterns. The complexity deepens with Murdica et al.’s demonstration that human sperm exposure to SPEVs enhanced tyrosine phosphorylation levels and promoted acrosome reaction [94]. Notably, Barranco et al. [95] and Tamessar et al. [96] reported null effects of SPEV treatment, finding neither alteration in capacitation status nor acrosome reaction in porcine and human sperm, respectively. The observed discrepancies may be attributed to multiple methodological variations, particularly the diversity of isolation methods that differentially impact both sample purity and yield, coupled with the inherent heterogeneity in SPEV morphology and molecular composition [97]. Additionally, biological variables including age, physiological status, and environmental conditions may act as confounding variables contributing to apparent discrepancies in experimental outcomes. Collectively, these findings elucidate the multifaceted roles of SPEV-contained proteins and ncRNAs in sperm motility and capacitation regulation. However, the potential interplay between these molecular cargoes and Ca^2+^-signaling pathways remains unexplored. Comprehensive investigations are needed to characterize the complex functional roles and regulatory networks through which SPEVs modulate critical sperm functions.

## 6. Function of Seminal Plasma Extracellular Vesicles in Female Reproductive Tract

Emerging evidence reveals that SPEVs play a critical role in mediating sperm survival, sperm-egg binding, and embryo implantation through modulating the immune system and establishing the endometrial receptivity during sperm transit in the FRT.

The FRT maintains a unique immunoregulatory microenvironment through sophisticated tolerance mechanisms. Successful fertilization and pregnancy critically depend on establishing immune tolerance toward allogeneic sperm and semi-allogeneic conceptus [98]. This specialized immunotolerant milieu is supported by distributed professional antigen-presenting cells (APCs) across the FRT, encompassing macrophages, DCs, and MHC class II-expressing epithelial populations. Once APCs encounter antigens, they process them internally and present fragments of these antigens on their surface using MHC molecules and activate cognate T-cells to differentiate into effector T-cells for pathogen clearance. However, within the unique immunologic milieu of the FRT, sperm- and conceptus-derived antigens are processed by APCs through a distinct mechanism. These APCs preferentially drive peripheral regulatory T cell (Treg) differentiation rather than effector T cell activation, thereby establishing active immune tolerance [99]. Disruption of the effector T cell/Treg equilibrium correlates with clinical manifestations ranging from impaired fertility and gestational complications to heightened infection susceptibility, reflecting the dual imperatives of FRT immunity: balancing pathogen defense with reproductive tolerance [100].

Notably, SPEVs have been demonstrated to induce the secretion of proinflammatory cytokines (particularly IL-6 and IL-8) [101,102], promote DC maturation [103], and subsequently drive naive T cells differentiating into Tregs [104]. Mechanistically, IL-6 serves as a pivotal mediator in pregnancy-related immune adaptation, where it not only regulates maternal–fetal immunological tolerance but also mediates critical processes during embryo implantation through orchestrating the targeted migration of trophoblast cells to the decidual interface [105]. Furthermore, IL-8 functions as a potent chemokine that recruits peripheral monocytes possessing the capacity to differentiate into DCs [106]. Compelling evidence from murine models reveals that depletion of uterine DCs during the implantation window results in disrupted vessel formation and subsequent embryo implantation failure [107]. In addition, as sperm migrate through the vagina, cervix, uterus, and oviduct, SPEVs attenuate natural killer (NK) cell-mediated cytotoxicity against sperm through CD48–CD244 receptor–ligand interaction, where CD48 molecules in SPEVs engage with the activating receptor CD244 expressed on NK cells, thereby shielding sperm from immune-mediated destruction [108]. These findings demonstrate that SPEVs modulate the establishment of immune tolerance, thereby preventing immune-related sperm damage and promoting embryo implantation. However, the molecular mechanisms underlying these processes remain to be elucidated and warrant further comprehensive investigation.

The establishment of receptive endometrium is essential for successful embryo implantation and pregnancy. Contemporary research estimates that suboptimal endometrial receptivity underlies approximately 67% of implantation failure cases, positioning it as the predominant etiological factor in recurrent implantation failure [109]. In 2014, Vojtech et al. characterized the small RNA expression profile of SPEVs, proposing their potential regulatory role in facilitating the establishment of endometrial receptivity prior to embryo implantation through regulating endometrial cell proliferation and inducing the expression of immune-related genes in the endometrium [19]. Subsequent studies have substantiated this regulatory paradigm. Notably, EVs derived from healthy donors’ seminal plasma significantly enhance endometrial receptivity through multiple mechanisms: (1) inducing in vitro decidualization of human endometrial stromal cells with concomitant prolactin secretion [110] and (2) upregulating receptivity-related molecular markers (MUC1, LIF, G-CSF, CX3CL1, VEGF) in endometrial epithelial cells [111]. Conversely, SPEVs from infertile patients exhibit inhibitory effects, suppressing both endometrial receptivity formation and marker gene expression [112]. Mechanistic studies further demonstrate that fertile SPEVs facilitate trophoblast–endometrial adhesion via LIF–STAT3 signaling pathway activation, ultimately promoting successful embryo implantation [113]. These findings collectively elucidate the crucial role of SPEVs in modulating the endometrial microenvironment for successful pregnancy establishment, providing novel insights into the molecular determinants of endometrial receptivity that may improve fertility outcomes.

## 7. Conclusions

Emerging evidence underscores the pivotal role of SPEVs in modulating reproductive systems. Although the precise molecular mechanisms through which SPEVs contribute to key reproductive processes including sperm maturation, sperm motility, sperm capacitation, establishment of immune tolerance, and receptive endometrium in the FRT remain elusive, these nanoscale vesicles present a new mode for fertility optimization via developing fertility assessment biomarkers and targeted modulation strategies. It is noteworthy that recent studies have highlighted the involvement of ncRNA-mediated ceRNA networks carried by SPEVs in mammalian reproductive regulation. In addition, SPEVs have been shown to modulate maternal uterine microenvironments through intercellular communication mechanisms. This discovery breaks through the traditional maternal–fetal dyadic regulatory paradigm central to embryo implantation studies, thereby unveiling novel transindividual regulatory dimensions in reproductive biology. Collectively, the findings summarized in this review not only advance our understanding through novel theoretical frameworks but also delineate critical methodological constraints and persistent knowledge gaps within this discipline. Future research directions should focus on the following:

(1) Integrating single-EV sequencing with Tissue EV isolation approaches to characterize SPEV subpopulations originating from distinct male reproductive glands, to elucidate novel regulatory pathways in mammalian reproductive systems.
(2) Leveraging contemporary advances in fertility biomarker discovery, conducting large-scale population validation studies to establish standardized fertility assessment tools, with particular emphasis on biomarkers that have been validated as functional regulators in mammalian reproductive systems.
(3) Systematically dissecting the ceRNA-mediated molecular crosstalk between SPEVs and reproductive tissues, ultimately leading to the discovery of precision therapeutic targets for fertility enhancement.
(4) Further investigating the molecular mechanisms through which SPEVs mediate modulation in uterine microenvironments during embryo implantation, providing novel insights for improving embryo implantation rates.

## Data Availability

No new data were created or analyzed in this study. Data sharing is not applicable to this article.

## References

[1] Zhang D., Wang Z., Luo X., Guo H., Qiu G., Gong Y., Gao H., Cui S. (2023). Cysteine Dioxygenase and Taurine Are Essential for Embryo Implantation by Involving in E2-ERα and P4-PR Signaling in Mouse. J. Anim. Sci. Biotechnol..

[2] Njagi P., Groot W., Arsenijevic J., Dyer S., Mburu G., Kiarie J. (2023). Financial Costs of Assisted Reproductive Technology for Patients in Low- and Middle-Income Countries: A Systematic Review. Hum. Reprod. Open.

[3] Niederberger C., Pellicer A., Cohen J., Gardner D.K., Palermo G.D., O’Neill C.L., Chow S., Rosenwaks Z., Cobo A., Swain J.E. (2018). Forty Years of IVF. Fertil. Steril..

[4] Bashiri Z., Amidi F., Amiri I., Zandieh Z., Maki C.B., Mohammadi F., Amiri S., Koruji M. (2021). Male Factors: The Role of Sperm in Preimplantation Embryo Quality. Reprod. Sci..

[5] Xie C., Huang C., Yan L., Yao R., Xiao J., Yang M., Chen H., Tang K., Zhou D., Lin P. (2024). Recipients’ and Environmental Factors Affecting the Pregnancy Rates of a Large, Fresh In Vitro Fertilization-Embryo Transfer Program for Dairy Cows in a Commercial Herd in China. Vet. Sci..

[6] Ahmadi H., Csabai T., Gorgey E., Rashidiani S., Parhizkar F., Aghebati-Maleki L. (2022). Composition and Effects of Seminal Plasma in the Female Reproductive Tracts on Implantation of Human Embryos. Biomed. Pharmacother..

[7] Pang P.-C., Chiu P.C.N., Lee C.-L., Chang L.-Y., Panico M., Morris H.R., Haslam S.M., Khoo K.-H., Clark G.F., Yeung W.S.B. (2011). Human Sperm Binding Is Mediated by the Sialyl-Lewis(x) Oligosaccharide on the Zona Pellucida. Science.

[8] Marlin R., Nugeyre M.-T., Tchitchek N., Parenti M., Lefebvre C., Hocini H., Benjelloun F., Cannou C., Nozza S., Dereuddre-Bosquet N. (2019). Seminal Plasma Exposures Strengthen Vaccine Responses in the Female Reproductive Tract Mucosae. Front. Immunol..

[9] Kalluri R., LeBleu V.S. (2020). The Biology, Function, and Biomedical Applications of Exosomes. Science.

[10] Ronquist G., Brody I., Gottfries A., Stegmayr B. (1978). An Mg^2+^ and Ca^2+^-Stimulated Adenosine Triphosphatase in Human Prostatic Fluid—Part II. Andrologia.

[11] van Niel G., D’Angelo G., Raposo G. (2018). Shedding Light on the Cell Biology of Extracellular Vesicles. Nat. Rev. Mol. Cell Biol..

[12] Johnstone R.M., Adam M., Hammond J.R., Orr L., Turbide C. (1987). Vesicle Formation during Reticulocyte Maturation. Association of Plasma Membrane Activities with Released Vesicles (Exosomes). J. Biol. Chem..

[13] Shamsi R.R., Jozani R.J., Asadpour R., Rahbar M., Taravat M. (2024). Seminal Plasma-Derived Exosome Preserves the Quality Parameters of the Post-Thaw Semen of Bulls with Low Freezeability. Biopreserv. Biobank..

[14] Zhang Z., Xu X., Chen F., Liu Q., Li Z., Zheng X., Zhao Y. (2025). Multi-Omics Sequencing Dissects the Atlas of Seminal Plasma Exosomes from Semen Containing Low or High Rates of Sperm with Cytoplasmic Droplets. Int. J. Mol. Sci..

[15] Yu K., Xiao K., Sun Q.-Q., Liu R.-F., Huang L.-F., Zhang P.-F., Xu H.-Y., Lu Y.-Q., Fu Q. (2023). Comparative Proteomic Analysis of Seminal Plasma Exosomes in Buffalo with High and Low Sperm Motility. BMC Genom..

[16] Leahy T., Rickard J.P., Pini T., Gadella B.M., de Graaf S.P. (2020). Quantitative Proteomic Analysis of Seminal Plasma, Sperm Membrane Proteins, and Seminal Extracellular Vesicles Suggests Vesicular Mechanisms Aid in the Removal and Addition of Proteins to the Ram Sperm Membrane. Proteomics.

[17] Carossino M., Dini P., Kalbfleisch T.S., Loynachan A.T., Canisso I.F., Shuck K.M., Timoney P.J., Cook R.F., Balasuriya U.B.R. (2018). Downregulation of MicroRNA Eca-Mir-128 in Seminal Exosomes and Enhanced Expression of CXCL16 in the Stallion Reproductive Tract Are Associated with Long-Term Persistence of Equine Arteritis Virus. J. Virol..

[18] Sakr O.G., Gad A., Cañón-Beltrán K., Cajas Y.N., Prochazka R., Rizos D., Rebollar P.G. (2023). Characterization and Identification of Extracellular Vesicles-Coupled miRNA Profiles in Seminal Plasma of Fertile and Subfertile Rabbit Bucks. Theriogenology.

[19] Vojtech L., Woo S., Hughes S., Levy C., Ballweber L., Sauteraud R.P., Strobl J., Westerberg K., Gottardo R., Tewari M. (2014). Exosomes in Human Semen Carry a Distinctive Repertoire of Small Non-Coding RNAs with Potential Regulatory Functions. Nucleic Acids Res..

[20] Rodriguez-Martinez H., Roca J., Alvarez-Rodriguez M., Martinez-Serrano C.A. (2022). How does the boar epididymis regulate the emission of fertile spermatozoa?. Anim. Reprod. Sci..

[21] Mancuso F., Calvitti M., Milardi D., Grande G., Falabella G., Arato I., Giovagnoli S., Vincenzoni F., Mancini F., Nastruzzi C. (2018). Testosterone and FSH Modulate Sertoli Cell Extracellular Secretion: Proteomic Analysis. Mol. Cell Endocrinol..

[22] Liang J., Mei J., Chen D., Xiao Z., Hu M., Wei S., Wang Z., Huang R., Li L., Ye T. (2024). The Role of Sertoli Cell-Derived miR-143-3p in Male Fertility Declines with Age. Mol. Ther. Nucleic Acids.

[23] Banijamali M., Höjer P., Nagy A., Hååg P., Gomero E.P., Stiller C., Kaminskyy V.O., Ekman S., Lewensohn R., Karlström A.E. (2022). Characterizing Single Extracellular Vesicles by Droplet Barcode Sequencing for Protein Analysis. J. Extracell. Vesicles.

[24] Neila-Montero M., Alvarez M., Riesco M.F., Soriano-Úbeda C., Montes-Garrido R., Palacin-Martinez C., de Paz P., Anel L., Anel-Lopez L. (2024). The Adaptation Time to the Extender as a Crucial Step for an Accurate Evaluation of Ram Sperm Quality during the Liquid Storage. Vet. Sci..

[25] Roca J., Broekhuijse M.L.W.J., Parrilla I., Rodriguez-Martinez H., Martinez E.A., Bolarin A. (2015). Boar Differences In Artificial Insemination Outcomes: Can They Be Minimized?. Reprod. Domest. Anim..

[26] Cannarella R., Condorelli R.A., Mongioì L.M., La Vignera S., Calogero A.E. (2020). Molecular Biology of Spermatogenesis: Novel Targets of Apparently Idiopathic Male Infertility. Int. J. Mol. Sci..

[27] Sung H., Ferlay J., Siegel R.L., Laversanne M., Soerjomataram I., Jemal A., Bray F. (2021). Global Cancer Statistics 2020: GLOBOCAN Estimates of Incidence and Mortality Worldwide for 36 Cancers in 185 Countries. CA Cancer J. Clin..

[28] Valadi H., Ekström K., Bossios A., Sjöstrand M., Lee J.J., Lötvall J.O. (2007). Exosome-Mediated Transfer of mRNAs and microRNAs Is a Novel Mechanism of Genetic Exchange between Cells. Nat. Cell Biol..

[29] Naqvi A.R., Slots J. (2021). Human and Herpesvirus microRNAs in Periodontal Disease. Periodontol. 2000.

[30] Dance A. (2024). Circular Logic: Understanding RNA’s Strangest Form Yet. Nature.

[31] Liu C.-X., Chen L.-L. (2022). Circular RNAs: Characterization, Cellular Roles, and Applications. Cell.

[32] Guo W., Ciwang R., Wang L., Zhang S., Liu N., Zhao J., Zhou L., Li H., Gao X., He J. (2024). CircRNA-5335 Regulates the Differentiation and Proliferation of Sheep Preadipocyte via the miR-125a-3p/STAT3 Pathway. Vet. Sci..

[33] Pal A., Karanwal S., Habib M.A., Josan F., Gaur V., Patel A., Garg M., Bhakat M., Datta T.K., Kumar R. (2025). Extracellular Vesicles in Seminal Plasma of Sahiwal Cattle Bulls Carry a Differential Abundance of Sperm Fertility-Associated Proteins for Augmenting the Functional Quality of Low-Fertile Bull Spermatozoa. Sci. Rep..

[34] Chauhan V., Kashyap P., Chera J.S., Pal A., Patel A., Karanwal S., Badrhan S., Josan F., Solanki S., Bhakat M. (2024). Differential Abundance of microRNAs in Seminal Plasma Extracellular Vesicles (EVs) in Sahiwal Cattle Bull Related to Male Fertility. Front. Cell Dev. Biol..

[35] Xu Z., Xie Y., Wu C., Gu T., Zhang X., Yang J., Yang H., Zheng E., Huang S., Xu Z. (2024). The Effects of Boar Seminal Plasma Extracellular Vesicles on Sperm Fertility. Theriogenology.

[36] Chen W., Xie Y., Xu Z., Shang Y., Yang W., Wang P., Wu Z., Cai G., Hong L. (2024). Identification and Functional Analysis of miRNAs in Extracellular Vesicles of Semen Plasma from High- and Low-Fertility Boars. Animals.

[37] Badrhan S., Karanwal S., Pal A., Chera J.S., Chauhan V., Patel A., Bhakat M., Datta T.K., Kumar R. (2024). Differential Protein Repertoires Related to Sperm Function Identified in Extracellular Vesicles (EVs) in Seminal Plasma of Distinct Fertility Buffalo (Bubalus Bubalis) Bulls. Front. Cell Dev. Biol..

[38] Ding N., Zhang Y., Wang J., Liu J., Zhang J., Zhang C., Zhou L., Cao J., Jiang L. (2025). Lipidomic and Transcriptomic Characteristics of Boar Seminal Plasma Extracellular Vesicles Associated with Sperm Motility. Biochim. Biophys. Acta Mol. Cell Biol. Lipids..

[39] Zhang Y., Ding N., Cao J., Zhang J., Liu J., Zhang C., Jiang L. (2024). Proteomics and Metabolic Characteristics of Boar Seminal Plasma Extracellular Vesicles Reveal Biomarker Candidates Related to Sperm Motility. J. Proteome Res..

[40] Zhao Y., Qin J., Sun J., He J., Sun Y., Yuan R., Li Z. (2024). Motility-Related microRNAs Identified in Pig Seminal Plasma Exosomes by High-Throughput Small RNA Sequencing. Theriogenology.

[41] Dlamini N.H., Nguyen T., Gad A., Tesfaye D., Liao S.F., Willard S.T., Ryan P.L., Feugang J.M. (2023). Characterization of Extracellular Vesicle-Coupled miRNA Profiles in Seminal Plasma of Boars with Divergent Semen Quality Status. Int. J. Mol. Sci..

[42] Ding Y., Ding N., Zhang Y., Xie S., Huang M., Ding X., Dong W., Zhang Q., Jiang L. (2021). MicroRNA-222 Transferred From Semen Extracellular Vesicles Inhibits Sperm Apoptosis by Targeting BCL2L11. Front. Cell Dev. Biol..

[43] Ding N., Zhang Y., Huang M., Liu J., Wang C., Zhang C., Cao J., Zhang Q., Jiang L. (2022). Circ-CREBBP Inhibits Sperm Apoptosis via the PI3K-Akt Signaling Pathway by Sponging miR-10384 and miR-143-3p. Commun. Biol..

[44] Huang J., Li S., Yang Y., Li C., Zuo Z., Zheng R., Chai J., Jiang S. (2024). GPX5-Enriched Exosomes Improve Sperm Quality and Fertilization Ability. Int. J. Mol. Sci..

[45] Sergeyev O., Bezuglov V., Soloveva N., Smigulina L., Denisova T., Dikov Y., Shtratnikova V., Vavilov N., Williams P.L., Korrick S. (2024). Intraindividual Variability of Semen Quality, Proteome, and sncRNA Profiles in a Healthy Cohort of Young Adults. Andrology.

[46] Oluwayiose O.A., Houle E., Whitcomb B.W., Suvorov A., Rahil T., Sites C.K., Krawetz S.A., Visconti P., Pilsner J.R. (2023). Altered Non-Coding RNA Profiles of Seminal Plasma Extracellular Vesicles of Men with Poor Semen Quality Undergoing in Vitro Fertilization Treatment. Andrology.

[47] Oluwayiose O.A., Houle E., Whitcomb B.W., Suvorov A., Rahil T., Sites C.K., Krawetz S.A., Visconti P.E., Pilsner J.R. (2023). Non-Coding RNAs from Seminal Plasma Extracellular Vesicles and Success of Live Birth among Couples Undergoing Fertility Treatment. Front. Cell Dev. Biol..

[48] Larriba S., Sánchez-Herrero J.F., Pluvinet R., López-Rodrigo O., Bassas L., Sumoy L. (2024). Seminal Extracellular Vesicle sncRNA Sequencing Reveals Altered miRNA/isomiR Profiles as Sperm Retrieval Biomarkers for Azoospermia. Andrology.

[49] Plata-Peña L., López-Rodrigo O., Bassas L., Larriba S. (2023). Experimental Validation of Seminal miR-31-5p as Biomarker for Azoospermia and Evaluation of the Effect of Preanalytical Variables. Andrology.

[50] Han X., Hao L., Shi Z., Li Y., Wang L., Li Z., Zhang Q., Hu F., Cao Y., Pang K. (2022). Seminal Plasma Extracellular Vesicles tRF-Val-AAC-010 Can Serve as a Predictive Factor of Successful Microdissection Testicular Sperm Extraction in Patients with Non-Obstructive Azoospermia. Reprod. Biol. Endocrinol..

[51] Yue D., Yang R., Xiong C., Yang R. (2022). Functional Prediction and Profiling of Exosomal circRNAs Derived from Seminal Plasma for the Diagnosis and Treatment of Oligoasthenospermia. Exp. Ther. Med..

[52] Chen H., Xie Y., Li Y., Zhang C., Lv L., Yao J., Deng C., Sun X., Zou X., Liu G. (2021). Outcome Prediction of Microdissection Testicular Sperm Extraction Based on Extracellular Vesicles piRNAs. J. Assist. Reprod. Genet..

[53] Panner Selvam M.K., Agarwal A., Sharma R., Samanta L., Gupta S., Dias T.R., Martins A.D. (2021). Protein Fingerprinting of Seminal Plasma Reveals Dysregulation of Exosome-Associated Proteins in Infertile Men with Unilateral Varicocele. World J. Mens. Health.

[54] Ma Y., Zhou Y., Xiao Q., Zou S.-S., Zhu Y.-C., Ping P., Chen X.-F. (2021). Seminal Exosomal miR-210-3p as a Potential Marker of Sertoli Cell Damage in Varicocele. Andrology.

[55] Zhang X., Vos H.R., Tao W., Stoorvogel W. (2020). Proteomic Profiling of Two Distinct Populations of Extracellular Vesicles Isolated from Human Seminal Plasma. Int. J. Mol. Sci..

[56] Chisholm J., Haas-Neill S., Margetts P., Al-Nedawi K. (2022). Characterization of Proteins, mRNAs, and miRNAs of Circulating Extracellular Vesicles from Prostate Cancer Patients Compared to Healthy Subjects. Front. Oncol..

[57] Zhang Y., Ding N., Xie S., Ding Y., Huang M., Ding X., Jiang L. (2021). Identification of Important Extracellular Vesicle RNA Molecules Related to Sperm Motility and Prostate Cancer. Extracell. Vesicles Circ. Nucl. Acids..

[58] Ferre-Giraldo A., Castells M., Sánchez-Herrero J.F., López-Rodrigo O., de Rocco-Ponce M., Bassas L., Vigués F., Sumoy L., Larriba S. (2024). Semen sEV tRF-Based Models Increase Non-Invasive Prediction Accuracy of Clinically Significant Prostate Cancer among Patients with Moderately Altered PSA Levels. Int. J. Mol. Sci..

[59] Conine C.C., Sun F., Song L., Rivera-Pérez J.A., Rando O.J. (2018). Small RNAs Gained during Epididymal Transit of Sperm Are Essential for Embryonic Development in Mice. Dev. Cell..

[60] Zhou W., De Iuliis G.N., Dun M.D., Nixon B. (2018). Characteristics of the Epididymal Luminal Environment Responsible for Sperm Maturation and Storage. Front. Endocrinol..

[61] Chen H., Pu L., Tian C., Qi X., Song J., Liao Y., Mo B., Li T. (2024). Exploring the Molecular Characteristics and Role of PDGFB in Testis and Epididymis Development of Tibetan Sheep. Vet. Sci..

[62] Aitken R.J., Nixon B., Lin M., Koppers A.J., Lee Y.H., Baker M.A. (2007). Proteomic Changes in Mammalian Spermatozoa during Epididymal Maturation. Asian J. Androl..

[63] Candenas L., Chianese R. (2020). Exosome Composition and Seminal Plasma Proteome: A Promising Source of Biomarkers of Male Infertility. Int. J. Mol. Sci..

[64] Simon C., Greening D.W., Bolumar D., Balaguer N., Salamonsen L.A., Vilella F. (2018). Extracellular Vesicles in Human Reproduction in Health and Disease. Endocr. Rev..

[65] Rejraji H., Sion B., Prensier G., Carreras M., Motta C., Frenoux J.-M., Vericel E., Grizard G., Vernet P., Drevet J.R. (2006). Lipid Remodeling of Murine Epididymosomes and Spermatozoa during Epididymal Maturation. Biol. Reprod..

[66] Kirchhoff C., Hale G. (1996). Cell-to-Cell Transfer of Glycosylphosphatidylinositol-Anchored Membrane Proteins during Sperm Maturation. Mol. Hum. Reprod..

[67] Miller D., Brinkworth M., Iles D. (2010). Paternal DNA Packaging in Spermatozoa: More than the Sum of Its Parts? DNA, Histones, Protamines and Epigenetics. Reproduction.

[68] Jones R. (1998). Plasma Membrane Structure and Remodelling during Sperm Maturation in the Epididymis. J. Reprod. Fertil. Suppl..

[69] Zhou W., Stanger S.J., Anderson A.L., Bernstein I.R., De Iuliis G.N., McCluskey A., McLaughlin E.A., Dun M.D., Nixon B. (2019). Mechanisms of Tethering and Cargo Transfer during Epididymosome-Sperm Interactions. BMC Biol..

[70] D’Amours O., Frenette G., Caron P., Belleannée C., Guillemette C., Sullivan R. (2016). Evidences of Biological Functions of Biliverdin Reductase A in the Bovine Epididymis. J. Cell Physiol..

[71] Caballero J.N., Frenette G., Belleannée C., Sullivan R. (2013). CD9-Positive Microvesicles Mediate the Transfer of Molecules to Bovine Spermatozoa during Epididymal Maturation. PLoS ONE.

[72] Sharma U., Conine C.C., Shea J.M., Boskovic A., Derr A.G., Bing X.Y., Belleannee C., Kucukural A., Serra R.W., Sun F. (2016). Biogenesis and Function of tRNA Fragments during Sperm Maturation and Fertilization in Mammals. Science.

[73] Reilly J.N., McLaughlin E.A., Stanger S.J., Anderson A.L., Hutcheon K., Church K., Mihalas B.P., Tyagi S., Holt J.E., Eamens A.L. (2016). Characterisation of Mouse Epididymosomes Reveals a Complex Profile of microRNAs and a Potential Mechanism for Modification of the Sperm Epigenome. Sci. Rep..

[74] Nixon B., De Iuliis G.N., Hart H.M., Zhou W., Mathe A., Bernstein I.R., Anderson A.L., Stanger S.J., Skerrett-Byrne D.A., Jamaluddin M.F.B. (2019). Proteomic Profiling of Mouse Epididymosomes Reveals Their Contributions to Post-Testicular Sperm Maturation. Mol. Cell Proteom..

[75] Yu Z.-L., Liu X.-C., Wu M., Shi S., Fu Q.-Y., Jia J., Chen G. (2022). Untouched Isolation Enables Targeted Functional Analysis of Tumour-Cell-Derived Extracellular Vesicles from Tumour Tissues. J. Extracell. Vesicles.

[76] Luo J., Zhu S., Kang Y., Liu X., Tan X., Zhao J., Ding X., Li H. (2024). Isolation of CD63-Positive Epididymosomes from Human Semen and Its Application in Improving Sperm Function. J. Extracell. Vesicles.

[77] Andrade A.F.C., Knox R.V., Torres M.A., Pavaneli A.P.P. (2022). What is the relevance of seminal plasma from a functional and preservation perspective?. Anim. Reprod. Sci..

[78] Hernández M., Roca J., Calvete J.J., Sanz L., Muiño-Blanco T., Cebrián-Pérez J.A., Vázquez J.M., Martínez E.A. (2014). Cryosurvival and in vitro fertilizing capacity postthaw is improved when boar spermatozoa are frozen in the presence of seminal plasma from good freezer boars. J. Androl..

[79] Heise A., Kähn W., Volkmann D.H., Thompson P.N., Gerber D. (2010). Influence of seminal plasma on fertility of fresh and frozen-thawed stallion epididymal spermatozoa. Anim. Reprod. Sci..

[80] Parrilla I., Martinez E.A., Gil M.A., Cuello C., Roca J., Rodriguez-Martinez H., Martinez C.A. (2020). Boar seminal plasma: Current insights on its potential role for assisted reproductive technologies in swine. Anim. Reprod..

[81] Schjenken J.E., Robertson S.A. (2020). The female response to seminal fluid. Physiol. Rev..

[82] Martinez C.A., Cambra J.M., Gil M.A., Parrilla I., Alvarez-Rodriguez M., Rodriguez-Martinez H., Cuello C., Martinez E.A. (2020). Seminal plasma induces overexpression of genes associated with embryo development and implantation in Day-6 porcine blastocysts. Int. J. Mol. Sci..

[83] Morgan H., Eid N., Khoshkerdar A., Watkins A. (2020). Defining the male contribution to embryo quality and offspring health in assisted reproduction in farm animals. Anim. Reprod..

[84] Park K.-H., Kim B.-J., Kang J., Nam T.-S., Lim J.M., Kim H.T., Park J.K., Kim Y.G., Chae S.-W., Kim U.-H. (2011). Ca^2+^ Signaling Tools Acquired from Prostasomes Are Required for Progesterone-Induced Sperm Motility. Sci. Signal..

[85] Zhang X., Liang M., Song D., Huang R., Chen C., Liu X., Chen H., Wang Q., Sun X., Song J. (2024). Both Protein and Non-Protein Components in Extracellular Vesicles of Human Seminal Plasma Improve Human Sperm Function via CatSper-Mediated Calcium Signaling. Hum. Reprod..

[86] Guo H., Chang Z., Zhang Z., Zhao Y., Jiang X., Yu H., Zhang Y., Zhao R., He B. (2019). Extracellular ATPs Produced in Seminal Plasma Exosomes Regulate Boar Sperm Motility and Mitochondrial Metabolism. Theriogenology.

[87] Naz R.K., Rajesh P.B. (2004). Role of Tyrosine Phosphorylation in Sperm Capacitation/Acrosome Reaction. Reprod. Biol. Endocrinol..

[88] Petrunkina A.M., Waberski D., Günzel-Apel A.R., Töpfer-Petersen E. (2007). Determinants of Sperm Quality and Fertility in Domestic Species. Reproduction.

[89] Publicover S., Harper C.V., Barratt C. (2007). [Ca^2+^]_i_ Signalling in Sperm—Making the Most of What You’ve Got. Nat. Cell Biol..

[90] Fraser L.R. (2010). The “Switching on” of Mammalian Spermatozoa: Molecular Events Involved in Promotion and Regulation of Capacitation. Mol. Reprod. Dev..

[91] Xie Y., Xu Z., Wu C., Zhou C., Zhang X., Gu T., Yang J., Yang H., Zheng E., Xu Z. (2022). Extracellular Vesicle-Encapsulated miR-21-5p in Seminal Plasma Prevents Sperm Capacitation via Vinculin Inhibition. Theriogenology.

[92] Pons-Rejraji H., Artonne C., Sion B., Brugnon F., Canis M., Janny L., Grizard G. (2011). Prostasomes: Inhibitors of Capacitation and Modulators of Cellular Signalling in Human Sperm. Int. J. Androl..

[93] Bechoua S., Rieu I., Sion B., Grizard G. (2011). Prostasomes as Potential Modulators of Tyrosine Phosphorylation in Human Spermatozoa. Syst. Biol. Reprod. Med..

[94] Murdica V., Giacomini E., Alteri A., Bartolacci A., Cermisoni G.C., Zarovni N., Papaleo E., Montorsi F., Salonia A., Viganò P. (2019). Seminal Plasma of Men with Severe Asthenozoospermia Contain Exosomes That Affect Spermatozoa Motility and Capacitation. Fertil. Steril..

[95] Barranco I., Spinaci M., Nesci S., Mateo-Otero Y., Baldassarro V.A., Algieri C., Bucci D., Roca J. (2024). Seminal Extracellular Vesicles Alter Porcine in Vitro Fertilization Outcome by Modulating Sperm Metabolism. Theriogenology.

[96] Tamessar C.T., Anderson A.L., Bromfield E.G., Trigg N.A., Parameswaran S., Stanger S.J., Weidenhofer J., Zhang H.-M., Robertson S.A., Sharkey D.J. (2024). The Efficacy and Functional Consequences of Interactions between Human Spermatozoa and Seminal Fluid Extracellular Vesicles. Reprod. Fertil..

[97] Veerman R.E., Teeuwen L., Czarnewski P., Gucluler Akpinar G., Sandberg A., Cao X., Pernemalm M., Orre L.M., Gabrielsson S., Eldh M. (2021). Molecular Evaluation of Five Different Isolation Methods for Extracellular Vesicles Reveals Different Clinical Applicability and Subcellular Origin. J. Extracell. Vesicles.

[98] Nederlof I., Meuleman T., van der Hoorn M.L.P., Claas F.H.J., Eikmans M. (2017). The Seed to Success: The Role of Seminal Plasma in Pregnancy. J. Reprod. Immunol..

[99] Robertson S.A., Care A.S., Moldenhauer L.M. (2018). Regulatory T Cells in Embryo Implantation and the Immune Response to Pregnancy. J. Clin. Investig..

[100] Huang N., Chi H., Qiao J. (2020). Role of Regulatory T Cells in Regulating Fetal-Maternal Immune Tolerance in Healthy Pregnancies and Reproductive Diseases. Front. Immunol..

[101] Bai R., Latifi Z., Kusama K., Nakamura K., Shimada M., Imakawa K. (2018). Induction of Immune-Related Gene Expression by Seminal Exosomes in the Porcine Endometrium. Biochem. Biophys. Res. Commun..

[102] Paktinat S., Hashemi S.M., Ghaffari Novin M., Mohammadi-Yeganeh S., Salehpour S., Karamian A., Nazarian H. (2019). Seminal Exosomes Induce Interleukin-6 and Interleukin-8 Secretion by Human Endometrial Stromal Cells. Eur. J. Obstet. Gynecol. Reprod. Biol..

[103] Wang D., Jueraitetibaike K., Tang T., Wang Y., Jing J., Xue T., Ma J., Cao S., Lin Y., Li X. (2021). Seminal Plasma and Seminal Plasma Exosomes of Aged Male Mice Affect Early Embryo Implantation via Immunomodulation. Front. Immunol..

[104] Zhang X., Greve P.F., Minh T.T.N., Wubbolts R., Demir A.Y., Zaal E.A., Berkers C.R., Boes M., Stoorvogel W. (2024). Extracellular Vesicles from Seminal Plasma Interact with T Cells in Vitro and Drive Their Differentiation into Regulatory T-Cells. J. Extracell. Vesicles.

[105] Prins J.R., Gomez-Lopez N., Robertson S.A. (2012). Interleukin-6 in Pregnancy and Gestational Disorders. J. Reprod. Immunol..

[106] Mor G., Aldo P., Alvero A.B. (2017). The Unique Immunological and Microbial Aspects of Pregnancy. Nat. Rev. Immunol..

[107] Blois S.M., Alba Soto C.D., Tometten M., Klapp B.F., Margni R.A., Arck P.C. (2004). Lineage, Maturity, and Phenotype of Uterine Murine Dendritic Cells throughout Gestation Indicate a Protective Role in Maintaining Pregnancy. Biol. Reprod..

[108] Tarazona R., Delgado E., Guarnizo M.C., Roncero R.G., Morgado S., Sánchez-Correa B., Gordillo J.J., Dejulián J., Casado J.G. (2011). Human Prostasomes Express CD48 and Interfere with NK Cell Function. Immunobiology.

[109] Craciunas L., Gallos I., Chu J., Bourne T., Quenby S., Brosens J.J., Coomarasamy A. (2019). Conventional and Modern Markers of Endometrial Receptivity: A Systematic Review and Meta-Analysis. Hum. Reprod. Update.

[110] Rodriguez-Caro H., Dragovic R., Shen M., Dombi E., Mounce G., Field K., Meadows J., Turner K., Lunn D., Child T. (2019). In Vitro Decidualisation of Human Endometrial Stromal Cells Is Enhanced by Seminal Fluid Extracellular Vesicles. J. Extracell. Vesicles.

[111] Gholipour H., Amjadi F.S., Zandieh Z., Mehdizadeh M., Ajdary M., Delbandi A.A., Akbari Sene A., Aflatoonian R., Bakhtiyari M. (2023). Investigation of the Effect of Seminal Plasma Exosomes from the Normal and Oligoasthenoteratospermic Males in the Implantation Process. Rep. Biochem. Mol. Biol..

[112] Gholipour H., Bakhtiyari M., Amjadi F.S., Mehdizadeh M., Aflatoonian R., Zandieh Z. (2022). Evaluation of the Effect of Seminal Plasma Exosomes from Unexplained Infertile Men on the Expression of Implantation-Related Genes. Hum. Reprod..

[113] Wang H., Lin Y., Chen R., Zhu Y., Wang H., Li S., Yu L., Zhang K., Liu Y., Jing T. (2024). Human Seminal Extracellular Vesicles Enhance Endometrial Receptivity Through Leukemia Inhibitory Factor. Endocrinology.

